# Patient care and access to clinical trials in gynaecological oncology: global implications of the early phase of the COVID-19 pandemic

**DOI:** 10.1007/s00404-024-07511-4

**Published:** 2024-06-05

**Authors:** Sara Nasser, Christina Fotopoulou, Murat Gültekin, Desislava Dimitrova, Esra Bilir, Gülhan Inci, Philippe Morice, Mansoor Raza Mirza, Antonio Gonzalez Martin, Jonathan Berek, Jalid Sehouli, Collaborating Groups: European Network for Gynaecological Oncology Trials (ENGOT), European Society for Gynaecological Oncology (ESGO), Gynecological Cancer Intergroup (GCIG), Pan-Arabian Research Society for Gynecological Oncology (PARSGO), North-East German Gynecological Oncology Society (NOGGO)

**Affiliations:** 1 Department of Gynecology and Gynecological Oncology, Charite Campus Virchow Klinikum, Augustenburgerplatz 1, 13353 Berlin, Germany; 2 Department of Gynecological Oncology, Queen Charlotte’s Hospital, Imperial College London, London, UK; 3 Department of Gynecology and Obstetrics, Hacettepe University Hospitals, Ankara, Turkey; 4 Department of Global Health, Koç University Graduate School of Health Sciences, Istanbul, Turkey; 5 Department of Obstetrics and Gynecology, University Hospitals Schleswig-Holstein, Campus Kiel, Kiel, Germany; 6 Institute Gustave Roussy, Villejuif, France; 7 Department of Oncology, Rigshospitalet, Copenhagen University Hospital, Copenhagen, Denmark; 8 Clinica Universidad de Navarra, Pamplona, Navarra Spain; 9 Stanford Women´S Cancer Center, Stanford University, Palo Alto, CA USA

**Keywords:** COVID-19, Pandemic, Gynaecologic oncology, Clinical trials, Healthcare disparities

## Abstract

**Purpose:**

Our prospective international survey evaluated the impact of the early phase of the COVID-19 pandemic on the management gynaecological malignancies from the multidisciplinary physicians’ perspective with particular focus on clinical infrastructures and trial participation.

**Methods:**

Our survey consisted of 53 COVID-related questions. It was sent to healthcare professionals in gynaecological oncology centres across Europe and Pan-Arabian region via the study groups and gynaecological societies from April 2020 to October 2020. All healthcare professionals treating gynaecological cancers were able to participate in our survey.

**Results:**

A total of 255 answers were collected from 30 countries. The majority (73%) of participants were gynaecological oncologists from university hospitals (71%) with at least an Intensive Care Unit with cardiopulmonary support available at their institutions. Most institutions continued to perform elective surgeries only for oncological cases (98%). Patients had to wait on average 2 weeks longer for their surgery appointments compared to previous years (range 0–12 weeks). Most cases that were prioritised for surgical intervention across all gynaecological tumours were early-stage disease (74%), primary situation (61%) and good ECOG status (63%). The radicality of surgery did not change in the majority of cases (78%) across all tumour types. During the pandemic, only 38% of clinicians stated they would start a new clinical trial. Almost half of the participants stated the pandemic negatively impacted the financial structure and support for clinical trials. Approximately 20% of clinicians did not feel well-informed regarding clinical algorithm for COVID-19 patients throughout the pandemic. Thirty percent stated that they are currently having trouble in providing adequate medical care due to staff shortage.

**Conclusion:**

Despite well-established guidelines, pandemic clearly affected clinical research and patientcare. Our survey underlines the necessity for building robust emergency algorithms tailored to gynaecological oncology to minimise negative impact in crises and to preserve access to clinical trials.

## What does this study add to the clinical work

**Table:** 

Our study reveals a noteworthy increase in gynaecologic cancer surgery waiting times and significant disruptions to ongoing gynaecologic oncology clinical trials during the COVID-19 pandemic. Our findings underscore the widespread impact on clinical research, emphasizing the need for adaptive strategies in healthcare practices among gynaecologic oncology community.	

## Introduction

Over the course of last years, the novel coronavirus pandemic (COVID-19) caused a global public health emergency that has impacted medical professionals, infrastructures and the care of patients with gynaecological malignancies. Moreover, during the first half of the year 2020 alone, more than 23,000 papers dealing with various aspects of COVID-19 were published. To this day, the far-reaching medical, socio-economic and psychological implications of this disease are still to be evaluated. The pandemic has resulted in severe disruptions of the standard clinical procedures, research and clinical trials in the multidisciplinary aspects of gynaecological oncology care. Increasing emphasis has been placed on patient triage challenges, including the distortion of already existing algorithms and the observed heterogeneity of followed procedures. Clinical trial participation is defined as a quality indicator for healthcare systems due to the fact that this influences prognosis [1]. Although patients with gynaecologic cancers had significant anxiety regarding the cancer progression, they preferred to continue their treatments according to the plan from pre-pandemic era [2]. Moreover, the patients with gynaecologic cancers expressed their trust in their physicians during the pandemic [3]. However, many of them were lacking data about the impact of the pandemic on clinical trial participation.

We conducted a joint survey of European Society for Gynaecological Oncology (ESGO), Gynecological Cancer Intergroup (GCIG), and Pan-Arabian Research Society for Gynecological Oncology (PARSGO) members, as well as European Network for Gynaecological Oncology Trials (ENGOT) groups to evaluate the initial impact of the COVID-19 pandemic on the management of patients with gynaecological malignancies from the multidisciplinary physicians’ perspective. The particular focus was on clinical infrastructures, trial participation and maintenance therapy. The survey was designed to capture the dynamic changes observed at the beginning and with the development of the pandemic to build robust emergency algorithms tailored to gynaecological oncology patients in the future.

## Methods

The study utilised a cross-sectional design facilitated through an anonymous web-based survey. The survey was sent to all healthcare professionals in gynaecological oncology affiliated with ESGO PARSGO GCIG and ENGOT from April 2020 to October 2020. All healthcare professionals treating women with gynaecological cancers in European and Pan-Arabian centres were encouraged to participate in the study. Since the questionnaire was only administered in English, non-English speaking practitioners were automatically excluded.

The anonymous, self-administered, online survey consisted of 53 COVID-related questions. The survey was created using an online survey tool (www.surveymonkey.com) and structured into the following domains: participants’ characteristics (characteristics of institutions, available resources and cancer treatment logistics), triaging techniques, implications of COVID-19 on status of clinical trials, and the implications of COVID-19 on treatment and follow-up of gynaecological cancers. All designed questions were in close-ended format for the exception those addressing continuous variables (e.g. years of experiences, mean waiting time, etc.). All questions were required with the option of selecting “Not applicable” to account for uncertainty. Also, some questions allowed multiple options to be selected. The questions, although not formally validated, were created based on the major COVID-related topics that were being addressed by the recommendations from the gynaecological oncology societies at the time. They were reviewed by all authors and adjusted accordingly.

An introductory paragraph was created explaining the aim of the survey prior to commencing the questions. The survey link was then sent via email to the members of the participating societies. To prevent redundancy of responses, participants were limited to only one response in terms of questionnaire completion.

In accordance with the journal’s guidelines, we will provide our data for independent analysis by a selected team by the editorial team for the purposes of additional data analysis or for the reproducibility of this study in other centres if such is requested.

### Statistical analysis

Collected data were reported as frequencies [*n* (%)] if categorical, or as means (± standard deviation) if continuous and normally distributed. All percentages were calculated with the denominator being the number of respondents to each single question. The dependent variable amongst all associations was whether participating centres were cancer centres or not. All statistical analyses were conducted on the Statistical Package for Social Sciences (SPSS) version 23 (SPSS Inc., Chicago, IL, USA).

## Results

A total of 255 participants from 4 international societies representing 30 different countries in the European and Middle East and North Africa regions completed the questionnaire. The completion rate amongst the respondents was 89%. The majority (*n* = 177, 71.7%) of participants were gynaecological oncologists from university hospitals (*n* = 162, 65.6%) with at least an Intensive Care Unit with cardiopulmonary support available at their institutions (188, 76.1%). Most respondents were highly experienced physicians (161, 65%) with more than 11 years of experience in their respective fields (mean ± SD; 16.9 years ± 9.9), and the majority were in consultant leadership positions (167, 67.6%). The participants were mostly affiliated with certified cancer centres (163, 66.0%) and the majority had work volumes beyond 30 treated cases per year for ovarian, cervical and endometrial cancers (49.8%, 31.1% and 59.0%, respectively) (Table 1).

**Table 1:** The characteristics of the participants

Parameter	*n* (%)
Type of institution	
University hospital	162 (65.6)
District general hospital	42 (17.0)
General practice	13 (5.3)
Private practice	23 (9.3)
Other	15 (6.1)
Cancer centre	9 (3.6)
Specialist hospital	4 (1.6)
Nursing home	1 (0.4)
Medical supplier	1 (0.4)
Certified cancer centre	
Yes	163 (66.8)
No	81 (33.2)
Level of ICU	
ICU with cardiopulmonary support	188 (76.1)
ICU with ECMO support	54 (21.9)
Intermediate care unit	52 (21.1)
None	5 (2.0)
Ovarian cancer cases treated yearly	
1–10	38 (16.2)
10–20	36 (15.3)
20–30	44 (18.7)
> 30	117 (49.8)
Cervical cancer cases treated yearly	
1–10	62 (26.5)
10–20	62 (26.5)
20–30	35 (15.0)
> 30	75 (32.1)
Endometrial cancer cases treated yearly	
1–10	22 (9.4)
10–20	29 (12.4)
20–30	45 (19.2)
> 30	138 (59.0)
Perform elective surgery	
Yes	155 (62.8)
No	66 (26.7)
NA	26 (10.5)
Type of elective surgeries performed	
Gynaecological cancer surgery	132 (96.4)
Benign surgery	5 (3.6)
Cases for gynaecological cancer surgery	
Curative	121 (49.0)
Palliative	39 (15.8)
Emergency	105 (42.5)
All indications	80 (32.4)
Diagnostic	1 (0.4)
None	1 (0.4)
Running clinics	
Gynaecological oncology	134 (54.3)
Second opinion	46 (18.6)
Follow-up	57 (23.1)
Therapy monitoring	54 (21.9)
Other	4 (1.6)
Colposcopy	2 (0.8)
Urgent appointments	1 (0.4)
Breaking bad news	1 (0.4)

*ECMO* extracorporeal membrane oxygenation, *ICU* intensive care unit, *NA* not announced

### Surgical interventions

During the pandemic period, most respondents continued to perform elective surgeries (155, 62.8%), almost exclusively for oncological cases (132, 96.4%). Of which, curative cases (121, 49.0%) and emergencies (105, 42.5%) were the most prioritised. Generally, there was a significant reduction in operative capacity during the pandemic period with one-third (61, 37.2%) of respondents stating a reduction between 75–100% of their operative capacities (combined benign and oncology cases). In addition, benign cases tended to be reduced between 75–100% in 45.4% of respondents whilst oncology cases saw a reduction within the range of 0–25% in 71.4% of participants.

On average, patients had to wait up to 5 weeks longer for their elective surgery appointments during the pandemic as compared to previous years (mean wait time in weeks ± SD; 5.2 ± 8.3). For patients requiring radiotherapy treatment, the mean waiting time was 4.5 ± 2.4 weeks, whilst patients subjected to radiochemotherapy had a mean waiting time of 9.6 ± 19.6 weeks.

**Fig. 1:**
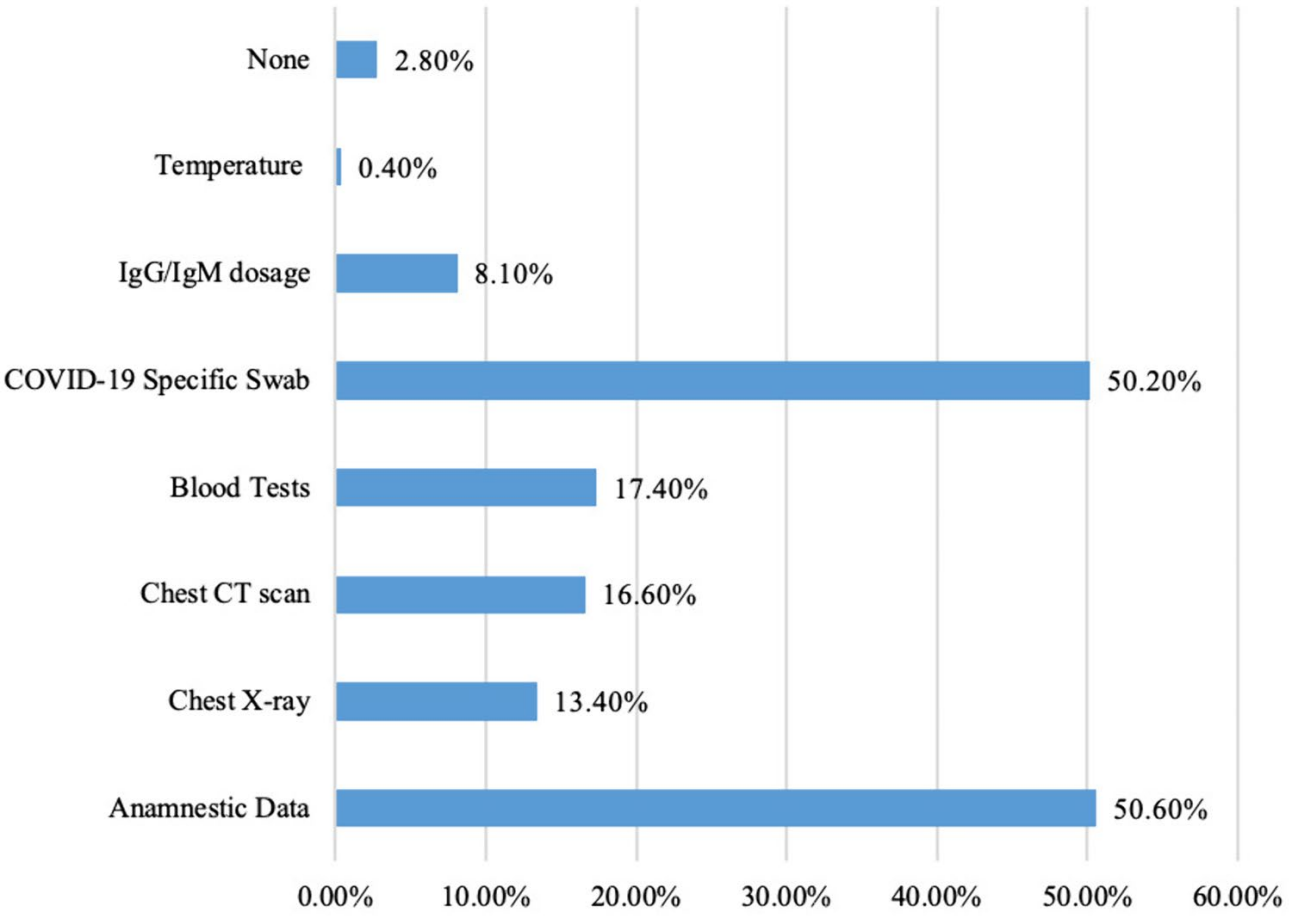
Triage techniques for patients with COVID-19 in the need of surgery

Amongst our combined cohort, the most adopted triaging techniques were anamnestic data (125, 50.6%), followed by COVID-19 swab tests (124, 50.2%). The use of serology was documented in only a minority of participants (20, 8.1%). Triage modalities utilised by respondents are demonstrated in Fig. 1. Patients positive for COVID-19 were frequently present in the institutions of 22.2% of respondents (*n* = 48). However, regular COVID-19 testing of healthcare staff was mostly conducted under symptomatic settings (101, 47.0%).

Shortages due to COVID-19 were pronounced amongst our respondents. Difficulties in providing medical care were demonstrated in 34.4% (*n* = 74). In addition, when operating on a patient with unknown COVID-19 status, classical surgical masks were utilised to conserve resources (71, 40.1%). Moreover, 34.1% of respondents did not have access to a separate operating room for patients tested positive with COVID-19. The majority of participants report that their respective institutions have developed SOPs for the perioperative management of gynaecological cancers in COVID-19 settings (104, 59.0%).

**Fig. 2:**
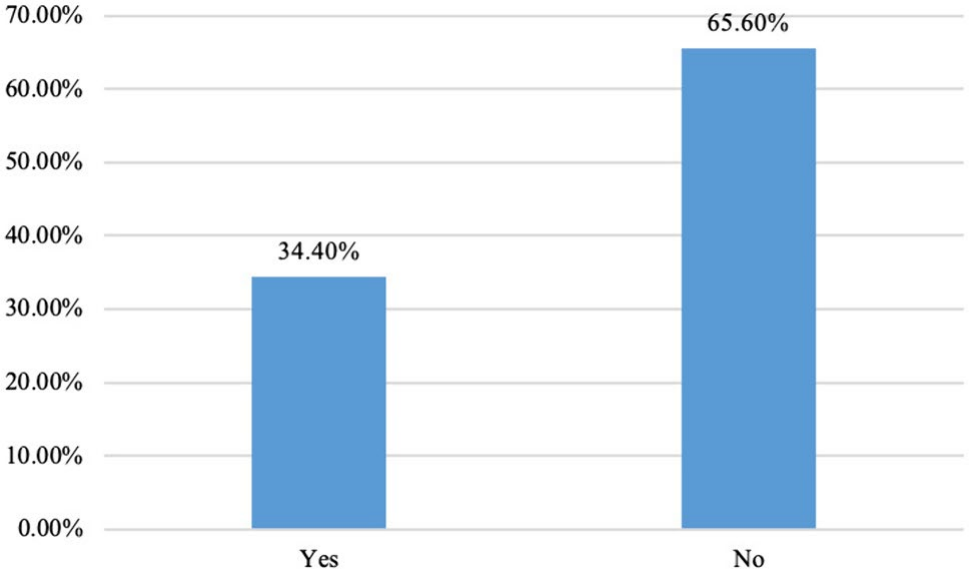
Difficulties in providing medical care due to staff shortage

When looking at the main gynaecological cancers treated amongst all participating centres, prioritisation of all cancer types (ovarian, cervical and endometrial) was given to primary cases in the early stages with good ECOG status (Fig. 2). Interestingly, 43.4% of clinicians stated they had changed their decision-making process regarding offering surgical treatment to their patients in light of the pandemic, as compared to 50.0% who had not changed their processes. About 51.0% of the surveyed respondents stated that in cases where surgery would be indicated, they were more likely to treat ovarian cancer cases with neoadjuvant chemotherapy instead of surgery as a result of the pandemic. This was not the case for endometrial or cervical cancer cases, where more than 70% of respondents stated they did not have an increased tendency to administer neoadjuvant treatment.

### Radiation therapy and radiochemotherapy

For patients requiring radiation therapy or radiochemotherapy, respondents stated only a minority of cases required active postponement of treatment considering the COVID-19 pandemic (15.8% and 10.8%, respectively). However, 20.5% respondents stated they did need to change or adapt the radiation regimens, in which fractionation was the aspect to be frequently altered (20, 55.6%). Regimen alteration was mostly adopted in cases with palliative intent (39, 15.8%), followed by cases with curative intent (27, 10.9%), and cases which require symptomatic control (26, 10.5%).

### Outpatient clinics

More than half of the respondents stated they continued to run their outpatient clinics as usual (142, 76.3%); of which, more than half of the running clinics were gynaecological oncology outpatient clinics (134, 54.3%). Patients were followed up, kept well-informed regarding guidelines and risks of COVID-19 mainly though telephone calls (160, 64.8%), E-mails (61, 24.7%) and video conferences (15.4%). Social media channels were not reported to be used at that initial phase.

### General information and triaging

About 62% of respondents expressed their concerns regarding triaging and prioritising patients for treatment during the pandemic period. Nearly 85.0% of participants stated that they did discuss these concerns within their internal team structures. Furthermore, approximately 76% of the entire cohort demonstrated lack of awareness with regard to the clinical pathways for patients with COVID-19 and gynaecological malignancies. Whilst most respondents were open to receive supplementary information or training through webinars (29, 11.7%), conferences (20, 8.1%) or tumour boards (19, 7.7%), about half of the entire cohort rejected the need to receive professional support to deal with the ethical issues associated with patient management during the COVID-19 pandemic.

### Clinical trials

More than half of the respondents experienced an overall reduction in their study and trial activities as compared to the pre-COVID-19 era (45, 54.9%). About 35.5% stated they continued to run ongoing clinical trials during the pandemic period, compared to 64.5% who stopped their clinical trials and 19.7% who halted recruitment of new patients. The majority also continued to provide maintenance therapy (including PARP inhibitors, bevacizumab and hormonal therapies as indicated) to their patients during the pandemic (194, 78.5%).

**Fig. 3:**
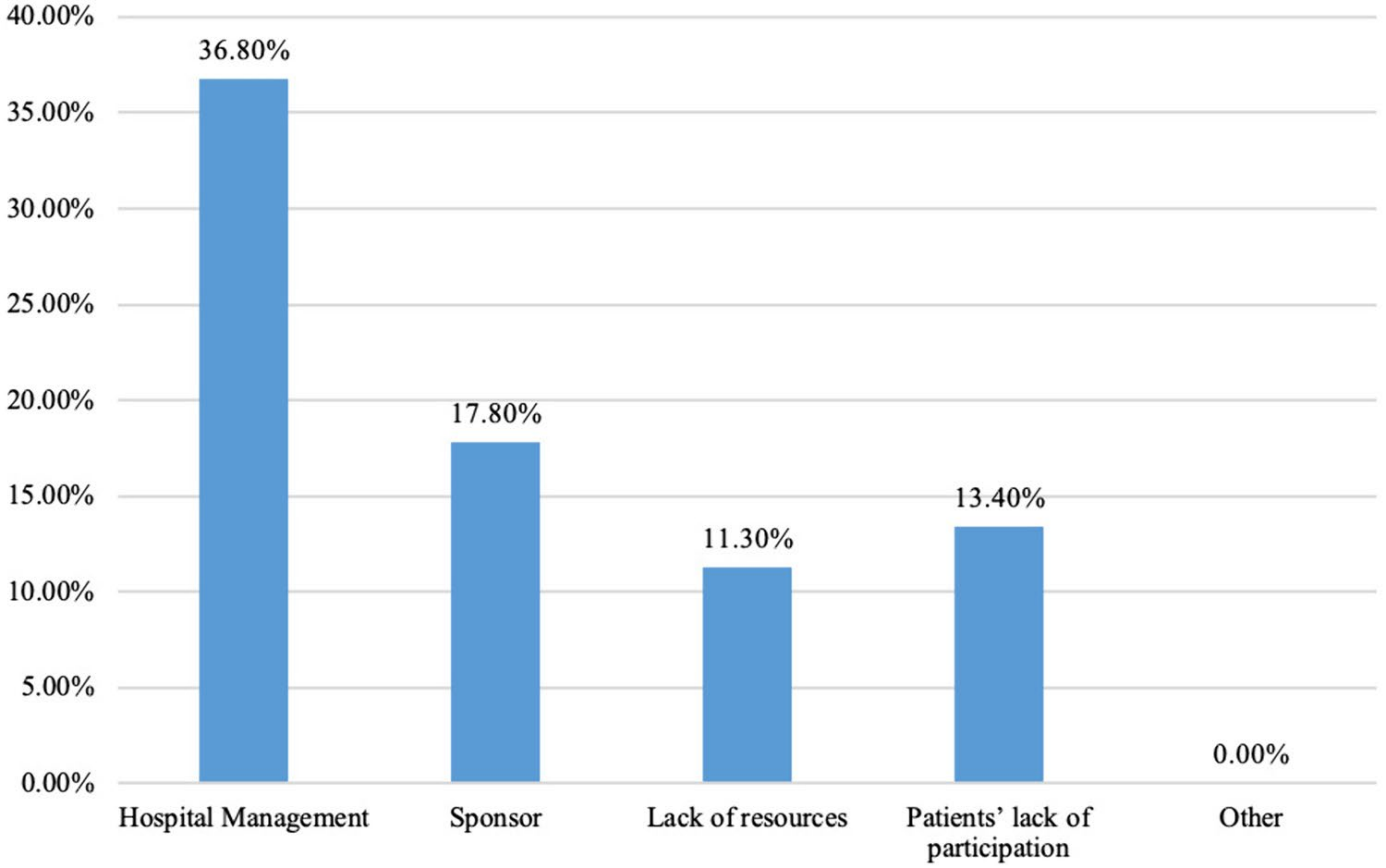
Influencers to reduce clinical trials activity

**Fig. 4:**
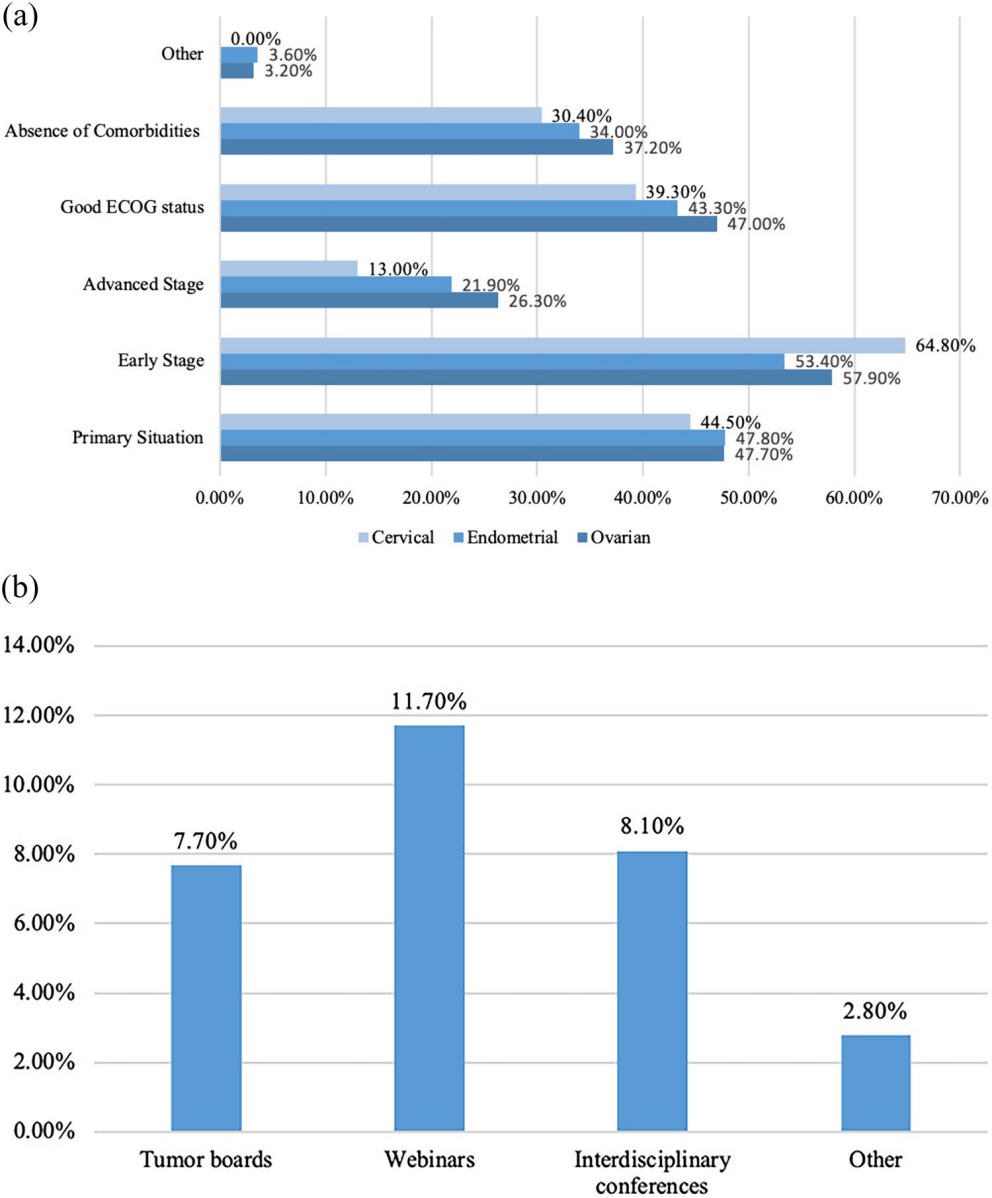
**A** Prioritisation of the gynaecologic oncology cases. **B** Preferences of the platforms to receive further information or training

The majority of the trials that continued to run during the pandemic were Phase III trials (Fig. 3). Respondents stated that the main criteria for prioritising which trials to continue running were trial costs and the size of the trial as shown in Fig. 4A.

About 47.3% disclosed that the pandemic has negatively impacted their financial structure and support for clinical trials. Of those, one quarter (21, 60.0%) evaluated the severity of this impact as being moderate. Subsequently, the majority stated they did not have relevant support from their central administrative bodies (51, 70.8%) or the government (57, 79.2%) to preserve their trial activities. In addition, governmental guidelines for the conduction of clinical trials within the COVID-19 era were severely lacking (28, 51.4%), Fig. 4B**.**

In terms of respondents’ practises to trials within a COVID-19 context, 20.3% routinely screened for COVID-19 patients. Most of which was oriented around Phase II and III clinical trials (13.4%, both). The presence of COVID-19 positive patients was reported by 18.9% of respondents (*n* = 14). In such cases, stopping or delaying the intervention was the most commonly adopted mitigation strategy (7, 50.0%).

## Discussion

The eruption of the COVID-19 pandemic immensely disturbed all healthcare delivery models [4]. Cancer clinicians have been greatly affected as COVID-19 has augmented cancer care uncertainties beyond its originally inherent margins. In addition, elective surgical procedures have undergone a rapid reduction justified by a safety intention to limit COVID-19 spread to either patients or healthcare workers and a logistic intention to preserve resources amidst an unprecedented surge of patients [5–7]. In light of the above, our survey demonstrates the heterogeneity of clinical practise and the impact of the early phase of the pandemic on clinical care and trial recruitment at an international level.

Initially the general consensus amongst the literature in terms of cancer care during the early phase of the pandemic was to postpone all forms of elective surgeries for benign lesion [6, 7]. Despite its potential interference with the cancer care continuum, such an approach was adopted to conserve resources and limit the spread of COVID-19 through mediums like hospitals [7]. Undeterred by the significant reductions in operative capacity amongst respondents of different oncologic societies, our survey demonstrated that the greater portion of gynaecologic oncologists provided elective surgeries, that were mostly of curative intent. Further heterogeneity can be observed within the variability of adopted triaging methods throughout our cohort and in between different published studies as there exists no consensus regarding optimal triaging methods [8–10]. Nonetheless, a compelling argument is that triaging, whilst needing to be guided by international cooperation of oncological bodies, should be dynamic with daily variations [11]. Whilst there is no “one size fits all” approach for cancer care amidst a rapidly growing pandemic, the implications of such heterogeneity manifests in the unnecessary expenditure of scarce resources as some respondents reported inability to provide equitable medical care, failure to provide maintenance therapy, shutting down of trials, and lack of adequate personal protective equipment.

Recent guidelines advocate for the limitation of patient–staff exposure and resource sustainability through the utilisation of surgical prioritisation guidelines [6]. Prioritisation is determined by the urgency of condition, resource availability, disease prevalence, patient and tumour characteristics and expected outcomes from delays, by which life-threatening conditions and aggressive tumours with a propensity for early metastasis attain the highest levels of urgency [7]. An international prospective cohort study including almost 5500 patients with colorectal cancer revealed that the delays due to the pandemic did not negatively affect resectability (OR 1.18, 95% CI 0.90–1.55, *p* = 0.224) [12]. However, another international prospective cohort study, including almost 4000 patients with gynaecologic cancers, reported significant adverse effects due to significantly delayed surgeries (> 8 weeks) (*p* = 0.024) [13]. Our survey shows that early cancer stage, primary situation and good ECOG status were the most prevalent criteria for prioritisation across cervical, endometrial and ovarian cancer management. Such adopted prioritisation is essentially the opposite of proposed guidelines, as only urgent/emergency cases should take the utmost of priority, followed by high grade/stage disease [14, 15]. On the other hand, early-stage disease can be delayed from 2 to 12 weeks depending on the type of cancer [15]. In fact, in areas with high prevalence of COVID-19, surgery for advanced-stage gynaecologic cancers can be postponed in lieu of chemotherapy [7]; a trend which was observed in the management of only ovarian cancer amongst our study respondents. These prioritisation algorithms were devised to shorten hospital stay, reduce postoperative complications, thus limiting patient exposure to the hospital environment [6].

Amongst our surveyed gynaecologic societies, the impact of COVID-19 on maintenance care of patients with cancer was manifested as delays in chemoradiotherapy, alterations in administrated doses or fractionation, increased waiting times reaching up to 10 weeks and shutdown of clinics. The adoption of nonstandard therapeutic regimens or altered follow-up scheduling was observed to significantly increase postoperative complications [6, 16]. Suspicions arise regarding such an approach as it could prove detrimental to patients’ health aggravating their performance status and potential loss of treatment window, most notably in those with metastatic disease [6, 17]. To mitigate these implications, alternative neoadjuvant treatments might be sought, the likes of PARP inhibitors or hormonal therapy [18]. In addition, telemedicine has been adopted by a variety of centres as virtual technology enables the conservation of PPE, reduces patient physician exposure and allows for the sustainability of clinical education [7]. Tele-surveillance models using patient-reported outcomes proved to be a reliable tool in modern patient care as it is associated with improved compliance, survival rate, shorter waiting time and reduced hospitalisation [19]. Moreover, this tool has the added benefit of facilitating proper and effective screening and triage whilst maintaining scheduled appointments.

Our results demonstrate that COVID-19 has impaired institutions’ abilities to conduct clinical trials. Trial priority was mostly set by trial type as Phase III clinical trials were the most prioritised amongst our survey. On a global scale, COVID-19 hampered ongoing research practises and redirected research-related resources towards the exploration of COVID-19 care [20]. Prospective trials were cancelled, enrolment was reduced by nearly 50%, and attrition was augmented since COVID-19 positive cases were removed from trials [18, 21, 22]. Given the impact of the pandemic, institutions quickly adapted through the formulation of flexible policies which included effective and safe protocol mitigations such as remote monitoring, telemedicine, remote SIV, transference of laboratory testing to local labs, halting the collection of unnecessary correlative data and prioritising the distributing of interventions/drugs to participants’ households [21–24]. The A Pan-European study of the European Network of Gynaecological Cancer Advocacy Groups (ENGAGe) reported that 96.5% of the patients who were already part of a clinical trial expressed continuing to participate in the clinical trials during the pandemic [2]. Recent guidelines advocate for the aforementioned in addition to the prioritisation of Tier 1 studies and considering the burden of COVID-19 when designing new trials [6, 25].

The consequences of the pandemic have urged the international consortium to rethink on how to optimise clinical trials to formulate robust and flexible adaptations that could render clinical research resilient to future pandemics [26]. Furthermore, the pandemic highlighted the critical roles of biomedical research in everyday care, mandated changes to research conduct that were able to increase the efficiency of the research enterprise, and offered the potential to explore the basis of research prioritisation and funding [20]. Nevertheless, the lack of consistent guidelines and variation in trial implementation may predispose to research imbalances amongst cohorts and ultimately instill bias [6]. Amongst our second cohort, a significant portion continued to start new trials and recruit new patients without any form of COVID-19 screening embedded into their studies’ protocols. Moreover, governmental recommendations were lacking. We speculate that such deviance is not a product of disregard of sound clinical practises but perhaps due to variation in local COVID-19 status, staff conditions or number of recruited patients.

Currently, a shift to minimally invasive surgery (laparoscopy or robotic surgery) when possible is recommended [6, 16, 27, 28], as it is associated with shortened recovery and hospitalisation periods and thus minimising the risk for a perioperative COVID-19 infection. Alternatives to the standard cytology screening [29], new fractionation systematics [30] and the question of novel care and multidisciplinary communicative models have also been risen during this pandemic [7, 18, 20]. During the pandemic, decrease in minimally invasive surgery was reported by less-experienced gynaecologic oncologists [31]. Similar to our survey, Dogan et al. also reported neoadjuvant chemotherapy preference over radical surgery [31]. The challenge of surgical protocols for COVID-19-positive tested patients [25, 32] and the long-term healthcare strategies [7] still remain a global call for further research. Overall, the decision to embark on any form of treatment must be made after coherent communications between physicians and patients, most notably regarding the risk of delaying treatment against the risk of COVID-19-related morbidity and mortality due to increased hospital stay [6]. Personalised, prognosis-focussed, and clear communication is desirable for patients and is able to mitigate their stress and uncertainty in face of both COVID-19, cancer, and their associated disruption of standard care [3, 4].

The strengths of our study lie within its scope of participants as it sampled a highly specialised and experienced pool of gynaecological oncologists from 4 major societies representing about 30 different countries spanning Europe, Middle East and North Africa. Our results should be taken with caution due to the following limitations. The study is designed as a one-time point questionnaire, hence not powered for cross-nation comparison and not validated by original data. In addition, considering the rapidly evolving dynamics of COVID-19, the responses and behaviours are constantly changing amongst centres throughout the period of the pandemic. The close-ended nature of our survey may have missed other impacts of the COVID-19 pandemic on gynaecology oncology research and practise. Moreover, some data may be subjected to recall bias due to the self-reporting nature of the questionnaire, primarily items concerned with estimated time. Finally, whilst the study did garner high response rates from European and Pan-Arabian gynaecology oncology societies, COVID-19’s impact on the areas these societies was not equal and so does their responses in a cumulative analysis. Despite all limitations, we believe that our results provide relevant information and should be used as a basis for the development of crisis algorithms to preserve access to clinical trials and to create specific programmes for both education and information for healthcare providers and patients.

In light of the extensive variation, which was recurrent throughout the literature in terms of cancer care and clinical trials implementation, consistent emergency algorithms are required to avoid such disparity. Institutions are required to collaborate to reintegrate surgery and research within the context of a pandemic. The development of systematic and objective criteria for the prioritisation of cases and trials is integral. In addition, international and multi-centric studies should be conducted to evaluate the efficacy of different triage methods and alternate treatment regimens for patients with gynaecologic cancers. Finally, programmes should devise proper interventions to target increased physician stress, professional trauma and burnout.

## Data Availability

The study data can be obtained upon request to the corresponding author.
